# Rheumatoid Arthritis Patients after Initiation of a New Biologic Agent: Trajectories of Disease Activity in a Large Multinational Cohort Study

**DOI:** 10.1016/j.ebiom.2016.08.024

**Published:** 2016-08-18

**Authors:** D.S. Courvoisier, D. Alpizar-Rodriguez, J.E. Gottenberg, M.V. Hernandez, F. Iannone, E. Lie, M.J. Santos, K. Pavelka, C. Turesson, X. Mariette, D. Choquette, M.L. Hetland, A. Finckh

**Affiliations:** a University Hospitals Geneva, Switzerland; b Strasbourg University Hospital, France; c Hospital Clinic of Barcelona, Spain; d Rheumatology Unit, University Hospital, Bari, Italy; e Diakonhjemmet Hospital, Oslo, Norway; f Rheumatology Research Unit, Instituto de Medicina Molecular, Lisbon, Portugal; g Institute of Rheumatology, Prague, Czech Republic; h Department of Clinical Sciences, Malmö, Lund University, Malmö, Sweden; i Department of Rheumatology, Skåne University Hospital, Malmö, Sweden; j Hôpitaux Universitaires Paris-Sud, Université Paris-Sud, France; k Institut de Rhumatologie de Montréal, CHUM, Canada; l The DANBIO registry Rigshospitalet, Glostrup, University of Copenhagen, Denmark; m Department of Clinical Medicine, University of Copenhagen, Denmark

**Keywords:** Abatacept, Rheumatoid arthritis, Disease activity, DAS28, Longitudinal data, Drug retention, Response rate

## Abstract

**Background:**

Response to disease modifying antirheumatic drugs (DMARDs) in rheumatoid arthritis (RA) is often heterogeneous. We aimed to identify types of disease activity trajectories following the initiation of a new biologic DMARD (bDMARD).

**Methods:**

Pooled analysis of nine national registries of patients with diagnosis of RA, who initiated Abatacept and had at least two measures of disease activity (DAS28). We used growth mixture models to identify groups of patients with similar courses of treatment response, and examined these patients' characteristics and effectiveness outcomes.

**Findings:**

We identified three types of treatment response trajectories: ‘gradual responders’ (GR; 3576 patients, 91·7%) had a baseline mean DAS28 of 4·1 and progressive improvement over time; ‘rapid responders’ (RR; 219 patients, 5·6%) had higher baseline DAS28 and rapid improvement in disease activity; ‘inadequate responders’ (IR; 103 patients, 2·6%) had high DAS28 at baseline (5·1) and progressive worsening in disease activity. They were similar in baseline characteristics. Drug discontinuation for ineffectiveness was shorter among inadequate responders (*p* *=* *0.03*), and EULAR good or moderate responses at 1 year was much higher among ‘rapid responders’ (*p* *<* *0.001*).

**Interpretation:**

Clinical information and baseline clinical characteristics do not allow a reliable prediction of which trajectory the patients will follow after bDMARD initiation.

## Highlights

• This study examined disease activity trajectories in a multinational cohort of 3898 rheumatoid arthritis patients.
• Growth mixture models identified three groups: gradual, rapid, and inadequate responders (GR: 91·7%, RR: 5·6%, IR: 2·6%).
• At baseline, groups were similar in demographic and clinical characteristics, and moderately different in function and disease activity.
• The groups had large difference in drug retention and in good or moderate response rate.
  Using nine national registries, this study of 3898 established RA patients initiating a new bDMARD identified distinct types of responders: gradual, rapid and inadequate responders. Neither socio-demographic nor clinical characteristics at baseline allowed the prediction of the type of response trajectory after treatment initiation, but effectiveness outcomes strongly differed, suggesting that these empirically derived subgroups have clinical relevance.
  As a major aim of precision medicine is to make anti-rheumatic therapy more personalized, the detection of responder types following initiation of a specific bDMARD underscores the need to find reliable predictors of trajectories to identify patients needing a distinct treatment strategy.

## 1 Introduction

The effect of disease modifying antirheumatic drugs (DMARDs) in rheumatoid arthritis (RA) on disease activity is generally presented using population means (Combe et al., 2015, Gabay et al., 2015, Littlejohn et al., 2015). The use of biologic DMARDs (bDMARD) has revolutionized the therapy of severe RA (Sanmarti et al., 2015). However, the response to treatment is heterogeneous, both to cDMARDs (Aga et al., 2015), and to the various bDMARD agents (Kiely, 2015). As a major aim in the new era of precision medicine is to make anti-rheumatic therapy more personalized, identifying and predicting distinct treatment responses trajectories to DMARDs has major implications for clinical practice. Studies (range n: 568–2752) focused on identifying types of patients with similar evolutions in disease activity (Siemons et al., 2014), physical activity (Demmelmaier et al., 2016), functional limitation (Norton et al., 2013), or psychological distress (Norton et al., 2011) and found subsets of patients with less favorable trajectories. The identification of predictors of response type trajectories could enable an early identification of patients needing a distinct treatment strategy.

In RA, disease activity measures are the main clinical outcome used by practitioners to appraise the evolution of RA (Finckh et al., 2007), to modify and adapt treatment, and to determine if patients have reached a state of low disease activity (Inoue et al., 2007) or remission (Mohammed et al., 2015). The Disease Activity Score based on 28 joints (DAS28) is a well-established instrument to assess disease activity (Prevoo et al., 1995).

A study of early RA patients (Siemons et al., 2014) (*n* = 568) found three types of trajectories during the first year after treatment initiation: the most frequent type (82·6% of patients) was a good responder group, the second type (14·1%) comprised patients with a slower response to treatment, and the third one was composed of a very small group (3·3%) of patients who showed no improvement after 1 year. However, the trajectories of disease activity in patients initiating a specific bDMARD or in patients with established disease have not been studied.

The aim of this study was to identify different types of trajectories in RA disease activity following the initiation of a new bDMARD and to examine the determinants of each responder type in a large multi-national observational cohort.

## 2 Materials and Methods

### 2.1 Study Design

This is a pooled analysis of data from nine national registries of RA patients: ARTIS (Sweden), ATTRA (Czech Republic), DANBIO (Denmark), GISEA (Italy), NOR-DMARD (Norway), ORA (France), REUMA.PT (Portugal), RHUMADATA (Canada), and SCQM (Switzerland), collected from 2006 to 2015. Each of the registries was approved by the local Ethics Committee and national guidelines for collection of informed consent form before enrolment in the study in accordance with the Declaration of Helsinki were followed. A more detailed description of the registries is available elsewhere (Curtis et al., 2010, Finckh et al., 2015). Inclusion criteria for this analysis were a diagnosis of RA, initiation of ABA treatment and at least two assessments of DAS28. The primary outcome to model response trajectories was the disease activity score based on 28 joints (DAS28) (Prevoo et al., 1995). Most registries used DAS28-ESR; when not available, we used the DAS28-CRP instead. All patients had either complete data for the DAS28-ESR or complete data for the DAS28-CRP. Almost all registries had some patients with missing DAS28-ESR data. The Italian registry, GISEA, was the only registry with only DAS28-CRP data.

In addition to the DAS28, we extracted demographic variables, BMI, smoking status, comorbidities, seropositivity according to rheumatoid factor or anti-citrullinated protein antibodies (ACPAs), disease duration, and disability as measured by the health assessment questionnaire (HAQ). We also extracted information about treatment, including number of past biologics treatment, conventional DMARD (cDMARD) and glucocorticosteroid therapy. We further computed ABA drug retention, which integrates both drug effectiveness and tolerance, ABA drug retention until stopping for ineffectiveness, reason for ABA discontinuation, EULAR good or moderate response rate at 6 months, one year, and two years (van Riel, 2014) corrected for drug discontinuation (Lundex) (Kristensen et al., 2006). ABA drug retention was defined as the time between drug initiation and last administration, plus one dispensation interval. Patients lost to follow-up were censored at the last registered visit.

### 2.2 Statistical Analysis

We used growth mixture models (GMM) to identify groups of patients with similar courses of treatment response, modeling time since beginning of treatment as polynomials with a linear, quadratic and cubic terms and derived empirically based trajectory subgroups. GMM are used to model unobserved types of evolution of disease activity over time. GMM estimate groups of patients in which the trajectories of DAS28 are similar within each trajectory type and different from the trajectories in the other types (Reineke and Seddig, 2011). To determine the optimal number of types (also called latent classes in growth mixture models terminology) of disease activity trajectories, we used two information criteria (Akaike's information criteria, and Bayesian information criteria), with lower value indicating a better fit of the model to the data.

We then examined the association of these groups with demographic-, disease-, and treatment-related covariates, as well as treatment effectiveness. We analyzed patients and disease characteristics at treatment initiation using standard descriptive statistics and Fisher exact test for categorical variables or Wilcoxon rank sum test for continuous variables. We ran a sensitivity analysis using probability-weighted regression with weights based on posterior probability of classification in each trajectory group. Data are presented as means (SD) or medians (interquartile ranges) depending on their distribution. ABA drug retention was analyzed by the Kaplan Meier method. Given the inherent differences between registries (Finckh et al., 2015), we tested for effect modification by registry using an interaction term between types of trajectories and national registry using a Cox proportional hazard model. Analyses were performed using R v3.2.4 (R foundation, Vienna, Austria) and the lcmm package (Proust-Lima et al., 2015).

## 3 Results

**Fig. 1:**
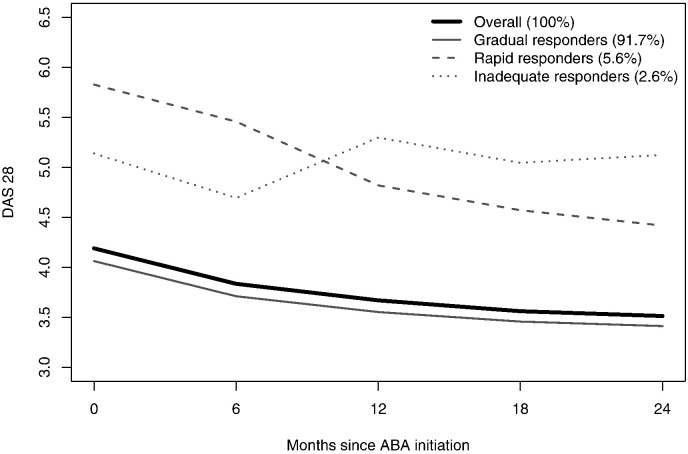
Class-specific DAS28 trajectories based on observed means. Time horizon was cut at 2 years to focus on the period with most available data.

A total of 3898 patients initiated ABA with a mean number of 3.94 DAS28 assessments. Follow-up time ranged from 1 month to 11.7 years. Trajectory analysis of the entire sample identified three types of disease activity trajectories with low misclassification (for goodness of fit indices, see Appendix 1). The largest group (3576 patients, 91·7%) can be labeled as the ‘gradual responders’ (GR) type, with a mean DAS28 at baseline of 4·1 and a progressive improvement over time. Fig. 1 presents the observed means for patients based on assigned types of trajectories. Estimated mean trajectories were quite similar (data not shown). The second group (219 patients, 5·6%) can be described as the ‘rapid responders’ (RR) type, with higher DAS28 values at baseline, and a rapid improvement in disease activity. The third group (103 patients, 2·6%) can be identified as ‘inadequate responders’ (IR) type, with higher DAS28 values at baseline, a short improvement during the first 6 months, followed by a return to initial disease activity level (for exact estimates of the DAS28 trajectories for these three types of patients, see Appendix 2).

**Fig. 2:**
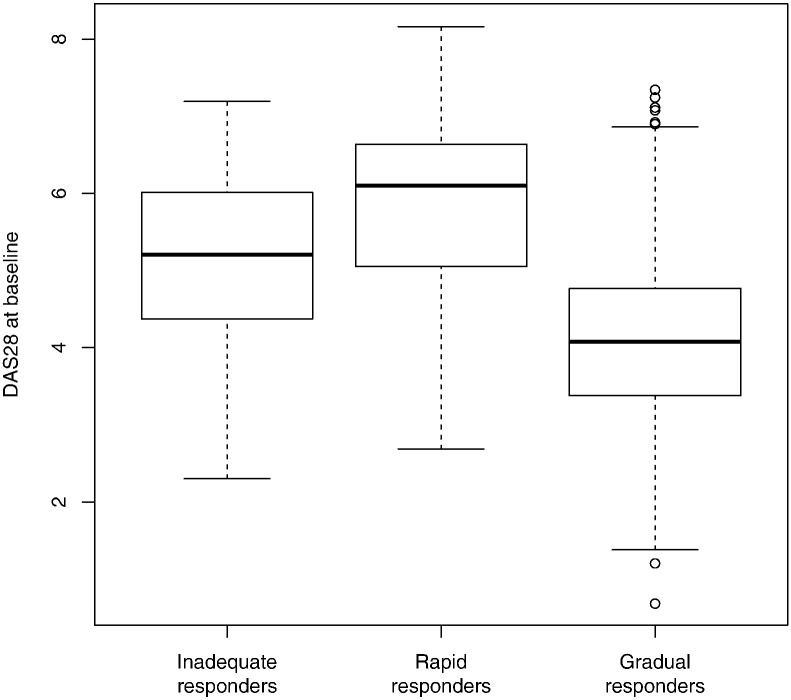
Boxplots of DAS28 values at baseline for the three types of trajectories.

The three types were similar in age, sex, BMI distributions, disease duration, and comorbidities (Table 1). ‘Gradual responders’ group had less disability at baseline (mean HAQ score: GR, 1·1; RR, 1·7; IR, 1·3, *p* < 0·001), and less previous treatment failures with cDMARDs and bDMARDs. Groups differed in mean DAS28 at baseline (p < 0·001), with ‘gradual responders’ generally presenting lower disease activity at baseline. However, these differences were not the main determinant of group membership since the variability of DAS28 at baseline was large, and there was a large overlap of DAS28 values between groups (Fig. 2). Groups also differed in the components of the DAS28 score (i.e., tender joints, swollen joints, ESR or CRP, and patient global assessment). The sensitivity analysis using probability-weighted regression accounting for uncertainty in classification of patients into three groups found similar results. In particular, significant and non-significant results remained the same.

ABA overall drug retention time was similar across all groups (*p* = 0·11). However, as could be expected, ABA drug retention until discontinuation for ineffectiveness was much shorter among ‘inadequate responders’ (median time in years: GR, 4·7, RR: 5·3, IR: 2·0, *p* *=* 0·03). The proportion of patients with EULAR good or moderate response rate (Lundex corrected) at 1 year was higher among ‘rapid responders’ (GR: 22·1%, RR: 39·2%, IR: 6·4%).

## 4 Discussion

Safety and efficacy of ABA in early and established RA has been demonstrated in several studies, using population means or – one could say – a single trajectory (Westhovens et al., 2009, Westhovens et al., 2014, Kremer et al., 2014, Schiff et al., 2011, Schiff et al., 2014). The present study focused on trajectory analyses of disease activity following the initiation of ABA, using growth mixture modeling to identify subgroups with similar response patterns. This study, which is a collaboration of nine national registries, is the first to analyze trajectories of disease activity in patients with established RA. Analysis of the entire sample identified three types of disease activity trajectories: a larger group of ‘gradual responders’ (91·7%), who improved gradually over time; a group of ‘rapid responders’ (5·6%), who started with a high DAS28 at baseline and improved quickly; and a smaller group of ‘inadequate responders’ (2·6%), who had a stable and relatively high disease activity over the first two years. Overall, socio-demographic and clinical characteristics at baseline were not strongly associated with future trajectory of disease activity after ABA treatment initiation. The importance of identifying these trajectories is reflected in the close association between clinical effectiveness and type of disease activity trajectory: The ‘inadequate responders’ discontinued ABA due to ineffectiveness much earlier compared to gradual and rapid responders. Furthermore, EULAR moderate or good response at 1 year was reached by almost none of the “inadequate responders”, compared to more than a third of the ‘rapid responders’.

Similarly to studies that examined disease activity trajectories in early RA (Barnabe et al., 2015, Siemons et al., 2014), we identified a large group of gradual responders and a small group of rapid responders. However, the present analysis also detected one group that displayed no improvement of their disease activity over time. The differences in findings could be due to study population or to the smaller sample size of previous studies. Whereas other studies focused on early RA patients on their first DMARD treatment, our analysis included more treatment resistant patients, often initiating a second or third line treatment, who often had long disease duration. In this difficult to treat patient group, it is not surprising to find inadequate responders, a subgroup probably composed of both primary non-responders and patients with secondary failures to this biologic agent. It is also possible that the smaller sample size and limited follow-up of other studies did not allow the detection of small trajectory subgroups.

In general, the patients in the three trajectory types could not be separated by baseline characteristics, except for higher disease activity and functional disability at baseline among rapid responders. This finding is in line with previous studies of patients with established RA showing that high DAS28 (Narvaez et al., 2016) and high HAQ score at baseline are associated with good response to bDMARD at 3 months (Kristensen et al., 2008). In contrast, studies of patients with early RA (i.e., with less chronicity) described a group of rapid responders with a lower DAS 28 at baseline, and found that patients' trajectory types differed in socio-demographic characteristics (e.g., sex, race, education) (Barnabe et al., 2015). The discrepancies in findings may be explained by differences in study population.

Much research is currently directed at identifying biomarkers to predict response and move towards personalized medicine; however no biomarkers have currently reached a level of discrimination to allow their use in clinical practice. Seropositivity for rheumatoid factor or anti-CCP antibodies has been consistently associated with a better effectiveness of ABA (Gottenberg et al., 2016), but were not associated with a specific disease activity trajectory in this analysis. Clinical effectiveness outcomes strongly differed between trajectories' types, in line with previous studies of disease activity or disability trajectories over time, in which type of trajectories was associated with mortality (Norton et al., 2013), remission (Siemons et al., 2014), or radiographic progression (Barnabe et al., 2015).

A limitation of this study is the observational nature of the data with the potential bias generated from attrition. In addition, unmeasured baseline characteristics, such as socioeconomic factors, may be associated with disease trajectories (Finckh et al., 2015). Another limitation is that DAS28 is a composite score, and the trajectories found in this study may not correspond to trajectories of the underlying scores. The strengths of this study include the large number of patients treated in a real-life setting, resulting from an international collaboration that allowed a pooled analysis of nine RA registries.

In conclusion, after ABA treatment initiation, different types of responders to treatment were identified: gradual, rapid and inadequate response groups, with differing drug discontinuation and response rates. However, clinical information such as seropositivity or disease duration, and baseline characteristics, do not allow to predict reliably the trajectory a patient will follow after ABA initiation. Other predictors of responder types should be explored to support clinical decision making.

## Funding

The study is investigator initiated and supported by an unrestricted research grant from Bristol Myers-Squibb. Funders had no role in study design, data collection, data analysis, interpretation and writing.

## Author Contribution

DSC did the data analysis, all authors contributed to data interpretation, provided comments on the manuscript writing, and approved the final manuscript.

## Figures and Tables

**Table:** 

Model	AIC	BIC	Percentage of total sample			
			Type 1	Type 2	Type 3	Type 4
						
M1. 1 class, random intercept	46,626.4	46,664.1	100			
M2. 1 class, random slope	46,424.0	46,467.8	100			
M3. 2 classes, random slope	46,269.5	46,344.7	80.2	19.8		
M4. 3 classes, random slope	46,163.0	46,269.6	91.7	5.6	2.6	
M5. 4 classes, random slope	52,549.8	52,713.0	82.8	11.1	4.5	1.6

**Table:** 

	Coefficient	*p*	95% CI
*Gradual responders*			
Intercept	4.06	< 0.001	3.92; 4.20
Time	− 0.69	< 0.001	− 0.88; − 0.50
Time^2^	0.28	< 0.001	0.19; 0.37
Time^3^	− 0.03	< 0.001	− 0.05; − 0.02

*Rapid responders*			
Intercept	5.83	< 0.001	5.47; 6.34
Time	− 2.63	< 0.001	− 3.32; − 1.94
Time^2^	0.81	< 0.001	0.47; 1.15
Time^3^	− 0.08	< 0.001	− 0.12; − 0.3

*Inadequate responders*			
Intercept	5.14	< 0.001	4,53; 5.75
Time	3.68	< 0.001	2.46; 4.90
Time^2^	− 1.51	< 0.001	− 2.09; − 0.93
Time^3^	0.17	< 0.001	0.09; 0.25

**Table 1:** Characteristics and outcomes of the patients in each of the three trajectories' type.

	‘Gradual Responders’	‘Rapid responders’	‘inadequate responders’	*p*
Number of patients, n (%)	3576 (91.7%)	219 (5.6%)	103 (2.6%)	
Time follow-up, years, mean (SD)	2.0 (1.7)	2.2 (2.2)	2.1 (1.6)	
Number of assessments, mean (SD)	3.9 (3.9)	5.0 (4.9)	4.5 (2.4)	
Age, years, mean (SD)	57.6 (13.0)	56.6 (12.7)	59.2 (12.6)	0.22
BMI, mean (SD)	25.8 (5.2)	26.1 (5.7)	28.1 (5.1)	0.10
Female, n (%)	2866 (80.1%)	187 (85.4%)	85 (82.5%)	0.14
Ever smoker, n (%)	582 (17.6%)	36 (17.2%)	14 (14.3%)	0.70
RF, n (%)	1521 (68.2%)	87 (64.4%)	47 (74.6%)	0.36
ACPA, n (%)	1165 (57.8%)	67 (51.5%)	41 (68.3%)	0.09
Disease duration, years, mean (SD)	12.5 (9.9)	11.9 (9.1)	12.7 (9.0)	0.63
HAQ, mean (SD)	1.1 (0.7)	1.7 (0.6)	1.3 (0.8)	< 0.001
Tender joints at baseline, mean (SD)	4.6 (5.3)	10.1 (7.1)	5.2 (6.6)	< 0.001
Swollen joints at baseline, mean (SD)	3.1 (3.7)	7.3 (6.2)	4.4 (4.4)	< 0.001
ESR at baseline, mean (SD)	23.3 (18.6)	36.2 (28.1)	32.1 (22.0)	< 0.001
CRP at baseline, mean (SD)	9.5 (16.1)	20.3 (32.7)	11.7 (16.0)	< 0.001
Patient global assessment, mean (SD)	47.5 (25.4)	61.5 (21.2)	45.0 (28.5)	< 0.001
DAS 28 at baseline, mean (SD)	4.1 (1.0)	5.8 (1.1)	5.1 (1.1)	< 0.001
N. past biologics, median [*IQR*]	1 [1; 2]	2 [1; 3]	2 [1; 3.5]	< 0.001
N past cDMARD, median [*IQR*]	2 [1; 4]	2.5 [1; 4]	3 [1; 4]	0.03
Glucocorticoids at baseline, n (%)	1773 (66.4%)	120 (67.4%)	66 (77.6%)	0.09
Comorbidities, n (%)				
Metabolic disease	47 (1.3%)	4 (1.8%)	0 (0.0%)	0.49
CV disease	80 (2.2%)	6 (2.7%)	2 (1.9%)	0.86
Infectious disease	35 (1.0%)	1 (0.5%)	0 (0.0%)	0.69
Cancer	8 (0.2%)	0 (0.0%)	0 (0.0%)	1.00
ABA drug retention^a^, years, median[95% CI]				
Overall	2.3 [2.1; 2.5]	1.6 [1.3; 3.0]	1.5 [1.4; 2.0]	0.11
Until ineffectiveness	4.7 [4.3; 5.1]	5.3 [2.6; −]	2.0 [1.7; −]	0.03
ABA discontinuation, n (%)	1981 (55.4%)	135 (61.6%)	78 (75.7%)	< 0.001
Reasons for discontinuation^b^, n (%)				0.17
For adverse events	333 (16.8%)	12 (8.9%)	9 (11.5%)	
For remission	19 (1.0%)	1 (0.7%)	0 (0.0%)	
For other reasons	483 (24.4%)	31 (23.0%)	20 (25.6%)	
For ineffectiveness	1146 (57.8%)	91 (67.4%)	49 (62.8%)	
EULAR moderate or good response				
At 6 months, n (%)	645 (24.3)	31 (21.2)	31 (32.9)	0.11
At 6 months, % Lundex	21.8	16.3	32.9	
At 1 year, n (%)	600 (30.8)	70 (66.0)	6 (8.2)	< 0.001
At 1 year, % Lundex	22.1	39.2	6.4	
At 2 years, n(%)	647 (54.9)	68 (94.4)	3 (10.0)	< 0.001
At 2 years, % Lundex	29.8	46.1	3.9	
Low disease activity (DAS28 < 3.2)				
At 6 months, N (%)	1120 (33.4)	15 (7.3)	16 (16.5)	< 0.001
At 1 year, N (%)	1256 (36.5)	36 (17.1)	1 (1.0)	< 0.001
At 2 years, N (%)	1433 (41.3)	54 (25.7)	1 (1.0)	< 0.001

a Estimated using Kaplan-Meier estimation.

b Other reasons include discontinuation due to pregnancy, surgery, and missing information on the specific reason for drug discontinuation.

## Appendix 1 Goodness-of-fit statistics for growth mixture modeling

For M4, Type 1 (Gradual responders), Type 2 (rapid responders), Type 3 (inadequate responders).

Posterior classification was 0.77% in Type 1, 0.81% in Type 2, and 0.94% in Type 3.
